# Reply to: Relevance of investigating light transmittance through red protective shields in dentistry

**DOI:** 10.1038/bdjopen.2018.5

**Published:** 2018-05-04

**Authors:** Vanida Nimmanon, Praewpat Pachimsawat, Siribang-on Pibooniyom Khovidhunkit, Bhornsawan Thanathornwong, Thirayost Nimmanon

**Affiliations:** 1 Department of Advanced General Dentistry, Faculty of Dentistry, Mahidol University, Bangkok, Thailand; 2 Department of General Dentistry, Faculty of Dentistry, Srinakharinwirot University, Bangkok, Thailand; 3 Department of Pathology, Phramongkutklao College of Medicine, Bangkok, Thailand

Thank you very much for giving us an opportunity to publish our work in this high quality and internationally renowned journal, BDJ Open, and for allowing us to respond to the letter from Dr Ellen Bruzell. We are very much grateful for Dr Ellen Bruzell’s expert opinions on our publication, titled ‘A study on light transmittance through red protective shields modified with different window films’.^1^ We did appreciate that LED-based light-curing dental devices have become increasingly popular because of their superior effectiveness over halogen-based light-curing units. Nevertheless, especially in developing countries, halogen-based light-curing units are still widely used in dental practice. Moreover, many of those light-curing units may not be up to the ISO standard possibly because of ageing or inadequate maintenance of the devices. These devices with filters that have deteriorated with time are therefore likely to emit high levels of radiation with undesired wavelengths, including red and near-infrared radiation, which may not be effectively filtered by protective shields that are currently used, resulting in heat-associated eye damage caused by the radiation exposure. Given the fact that light-curing dental devices have now been employed in a vast variety of dental practices, such as operative, orthodontic, paedodontic, aesthetic, or prosthodontic treatments, many of the dentists, either general practitioners or specialists, and other staff members who are working with the devices several hours per day are inevitably at risk of this radiation-associated danger. Without realisation of this danger, some staff members in dental clinics may even expose to the radiation without any protection. Even though ideally the use of LED-based or standardised halogen-based light-curing dental devices is highly encouraged, it may not be possible for numerous small dental clinics or hospitals in developing countries in soon time because of the costs of the devices. We therefore do believe that our published paper not only introduces an easy, affordable and practical way that is applicable to any dental clinics in the world for preventing this preventable danger, but also increases the awareness of this danger among dental personnel.
